# The Impact of Motor Imageries on Aesthetic Judgment of Chinese Calligraphy: An fMRI Study

**DOI:** 10.3389/fnhum.2021.706425

**Published:** 2021-08-06

**Authors:** Mingcheng He, Wei Zhang, Hira Shahid, Yushan Liu, Xiaoling Liang, Yan Duan, Hua Wang, Xianyou He

**Affiliations:** ^1^Key Laboratory of Brain, Cognition and Education Sciences, South China Normal University, Ministry of Education, Guangzhou, China; ^2^School of Psychology, South China Normal University, Guangzhou, China; ^3^Center for Studies of Psychological Application, South China Normal University, Guangzhou, China; ^4^Guangdong Key Laboratory of Mental Health and Cognitive Science, South China Normal University, Guangzhou, China; ^5^School of Fine Arts, South China Normal University, Guangzhou, China

**Keywords:** Chinese calligraphy, aesthetic judgment, kinesthetic imagery, visual imagery, fMRI

## Abstract

Previous behavioral studies on aesthetics demonstrated that there was a close association between perceived action and aesthetic appreciation. However, few studies explored whether motor imagery would influence aesthetic experience and its neural substrates. In the current study, Chinese calligraphy was used as the stimuli to explore the relationship between the motor imagery and the aesthetic judgments of a participant using functional magnetic resonance imaging. The imaging results showed that, compared with the baseline, the activation of the brain regions [e.g., anterior cingulate cortex (ACC), putamen, and insula] involved in perceptual processing, cognitive judgments, aesthetic emotional, and reward processing was observed after the participants performed motor imagery tasks. The contrast analyses within aesthetic judgments showed that the kinesthetic imagery significantly activated the middle frontal gyrus, postcentral gyrus, ACC, and thalamus. Generally, these areas were considered to be closely related to positive aesthetic experience and suggested that motor imagery, especially kinesthetic imagery, might be specifically associated with the aesthetic appreciation of Chinese calligraphy.

## Introduction

The creation of an artwork requires motor activity (Bullot, 2009; Ticini et al., 2014). Previous studies have revealed that, in pictorial art, the creative action of the artist could be imagined by observers automatically through different clues, such as the brushstrokes on the canvas or the traces on the sculpture (Taylor et al., 2012; Sbriscia-Fioretti et al., 2013; Ticini et al., 2014; Hoenen et al., 2017). In this study, for clarity, we defined this process of creative action perceived through the clues on the surface of the artwork as automatic imagery, which involves the simulation of an internal action (Stefan et al., 2013; Katinka and Lysanne, 2015). According to the theory of empathetic responses, Freedberg and Gallese (2007) believed that the activation of embodied mechanisms encompassing the simulation of actions (e.g., brushstrokes), emotions, and corporeal sensation consisted of the crucial element of aesthetic response, because these basic levels of reaction to artworks were essential to the understanding of the pictorial art. Also, the theory of visual imagination, proposed by Walton (1990), provided another possible explanation. Walton believed that the marks on the perceptible surface of a work of fine art function to trigger imaginative events that are partially constitutive of the perceptual content of that work. For example, oil painting is a planar perceptual stimulus of a two-dimensional pattern, so observers could perceive the painting as realistic three-dimensional scenes by the imagery (Seeley, 2006). In other words, the perceptual communications of observers with artworks could be increased by imagination. So, both the empirical studies and theoretical perspectives have suggested that the perception of the creative action of an artist plays an important role in the aesthetic experience of the observers. However, whether perceived creative action through active motor imagery would influence aesthetic experiences remains unclear.

Different from the automatic imagery, the creative action perceived through the active motor imagery guided by the instructions may involve a deeper processing or interaction with the objects. In this study, the authors defined the imagination of the creative action based on the specified instructions as motor (active) imagery. For example, Krishna et al. (2014) found that active motor imagery boosted the physiological responses of the consumers (e.g., salivating). Keesman et al. (2016) manipulated motor imagery through instructions and found that the participants who had guidelines of imaging the experience of eating food increased salivation, compared with those who only saw the food. Steinmetz et al. (2018) required the participants to imagine themselves in a very cold/warm environment and reported their current feelings. The results suggested that the participants in the cold/warm condition preferred more warming/cooling activities. These studies showed that people who actively imaged a given state developed more extra feelings parallel to the real experience or shifted the preferences of the people for relevant activities. In other words, spectators could mentally simulate an experience by imagining it in detail and evoke the same consequences as actually experiencing it, which might additionally improve their perceptual communications with the activity. The phenomenon may also be true for art aesthetics. Moreover, it should also be noted that the imagination of a creative action evoked by some clues (e.g., traces and brushstrokes) was unconscious or automatic processing. Since such ability still varied from person to person, it was much easier for those who were imaginative or who worked in the art field to automatically imagine the creative action (Milton et al., 2008). For example, observers with more ballet experience would show more activity in the facial muscle when they watched their favorite ballet moves, showing that they were more likely to habitually imagine these moves (Kirsch et al., 2016). Therefore, we were interested in finding whether active motor imagery affects the aesthetic experience of a participant. In the present study, we used an automatic imagery task as the baseline condition (BC) and further hypothesized that active motor imagery could influence the aesthetic experience of the participants compared with the BC. Moreover, active motor imagery contains kinesthetic imagery and visual imagery, which establish the subjective “distance” between the self and his/her own imaginal experience and distinguish the kinesthetic imagery from visual imagery (Jeannerod, 2011). Kinesthetic imagery requires people to feel the movement, to perceive the muscle contractions, and to stretch mentally from the (internal) perspective of the first person, which corresponds to the representation of a movement as if the individual takes part in the action himself/herself, whereas visual imagery requires self-visualization of a movement from the (external) perspective of the third person, which corresponds to the representation of the movement as if the subject is a spectator and that somebody (i.e., himself or another person) performs the action (Guillot et al., 2009). So, compared with visual imagery, kinesthetic imagery may have a closer subject distance and is easier to perform. According to the aesthetic fluency theory proposed by Reber et al. (2004), positive aesthetic experiences are linked to processing ease. Therefore, we further hypothesized that, compared with visual imagery, kinesthetic imagery of the creative action may contribute better to the positive aesthetic judgments of the participants than the BC.

In addition, Chinese calligraphy is an important practice of symbolic art in China, which particularly stresses the creative action of the artist (Xu et al., 2016), and contains the specific writing of each stroke, and the writing rhythm (Chen et al., 2017). Furthermore, critics of traditional calligraphy mainly stress the significance of the imagery of the artistic action of a calligrapher about the aesthetics of calligraphy. They believed that the imagery of calligraphy formation is a process of artistic recreation, which could help viewers to appreciate the beauty of calligraphy. For example, Jiang Kui, a famous calligraphy critic in the Song Dynasty of China (1127–1279 AD), pointed out that when he appreciated famous calligraphy artworks, even he could imagine the action of the creator of the calligraphy artwork as if the creator was visible there. In one word, Chinese calligraphy is a suitable artistic medium to examine the influence of motor imagery on aesthetic experience.

This study aims to investigate whether active motor imagery of the creative action of an artist is more likely to influence the aesthetic experience of an observer than the BC. Chinese calligraphy was used as the material to explore the relationship between active motor imagery and the aesthetic judgments of a participant through functional magnetic resonance imaging (fMRI). If active motor imagery would affect aesthetic judgments, it can be predicted that the significant activation in motor-related regions such as the parietal lobe after the participants perform the different motor imagery tasks about the creative actions of creators. Several studies have reported that the premotor cortex and the superior parietal lobule were significantly activated during motor imagery (see meta-analysis by Hardwick et al., 2018). Meanwhile, previous studies revealed that multiple functions were involved in aesthetic judgments, including perceptual processing, cognitive judgment, aesthetic emotional, and rewarding processing (see meta-analysis by Boccia et al., 2016; Hu et al., 2020). Many imaging studies have found that the perceptual processing of beauty is associated with the activities in the occipital lobe such as the middle occipital gyrus and the fusiform gyrus, regions that involve visual preference and attention modulation. Cognitive judgments of beauty are associated with activation in the frontal lobe such as the middle and the inferior frontal gyrus. Emotional and rewarding processing are major components for aesthetic judgments and could guide motivation and decision-making (Vartanian and Skov, 2014; Zhang et al., 2019). For example, the imaging results in Zhang et al. (2017) revealed that the regions associated with perceptual, cognitive, emotional, and rewarding processing are commonly activated in beautiful judgments of both pictographs and oracle bone scripts. Therefore, if the processing of motor imagery indeed contributes to the aesthetic experience of an observer, not only the activation of brain regions associated with perception and judgment processing but also the activation of emotional and rewarding processing regions, such as the anterior cingulate cortex (ACC), the putamen cortex, and the insula (Boccia et al., 2016; Di Dio et al., 2016; Skov and Nadal, 2020), which relate to aesthetic judgments, should be observed.

## Method

### Participants

We recruited 20 healthy right-handed University students (i.e., 8 men and 12 women), aged between 19 and 24 years (*M*_age_ = 21.7, *SD* = 2.43), for this experiment. All participants had a normal or corrected-to-normal vision. None of them had received professional training in the art. Written informed consent was obtained from each participant, and the protocol was approved by the Institute Ethics Committee, South China Normal University, Guangdong, China.

### Experimental Procedures

#### Stimuli

The stimuli consisted of 72 images of brush-pen calligraphy. First, 72 Chinese characters were picked up from the Chinese national corpus (http://www.aihanyu.org/cncorpus/index.aspx), and the word frequency range is 0.7822–0.0002%. Second, a proficient calligraphist was invited to write these 72 characters for calligraphy images. Later, all the images were randomly assigned into three groups, namely, kinesthetic imagery, visual imagery, and BC, with 24 images in each group. To control the homogeneity of the three groups of materials, another 35 participants (i.e., 9 men and 26 women, age ranging from 17 to 29 years) used a seven-point (values from 1 to 7) scale to rate the complexity, familiarity, valence, frequency, imaginability, and beauty of the characters. No significant statistical difference was found in complexity [*F*_(2, 69)_ = 0.37, *p* = 0.691], familiarity [*F*_(2,69)_ = 1.19, *p* = 0.315], valence [*F*_(2,69)_ = 0.38, *p* = 0.108], and beauty [*F*_(2,69)_ = 1.39, *p* = 0.261], imaginability [*F*_(2,69)_ = 0.14, *p* = 0.86], as well in frequency [*F*_(2,69)_ = 1.177, *p* = 0.31] among the three groups of materials.

### Procedure

**Figure 1:**
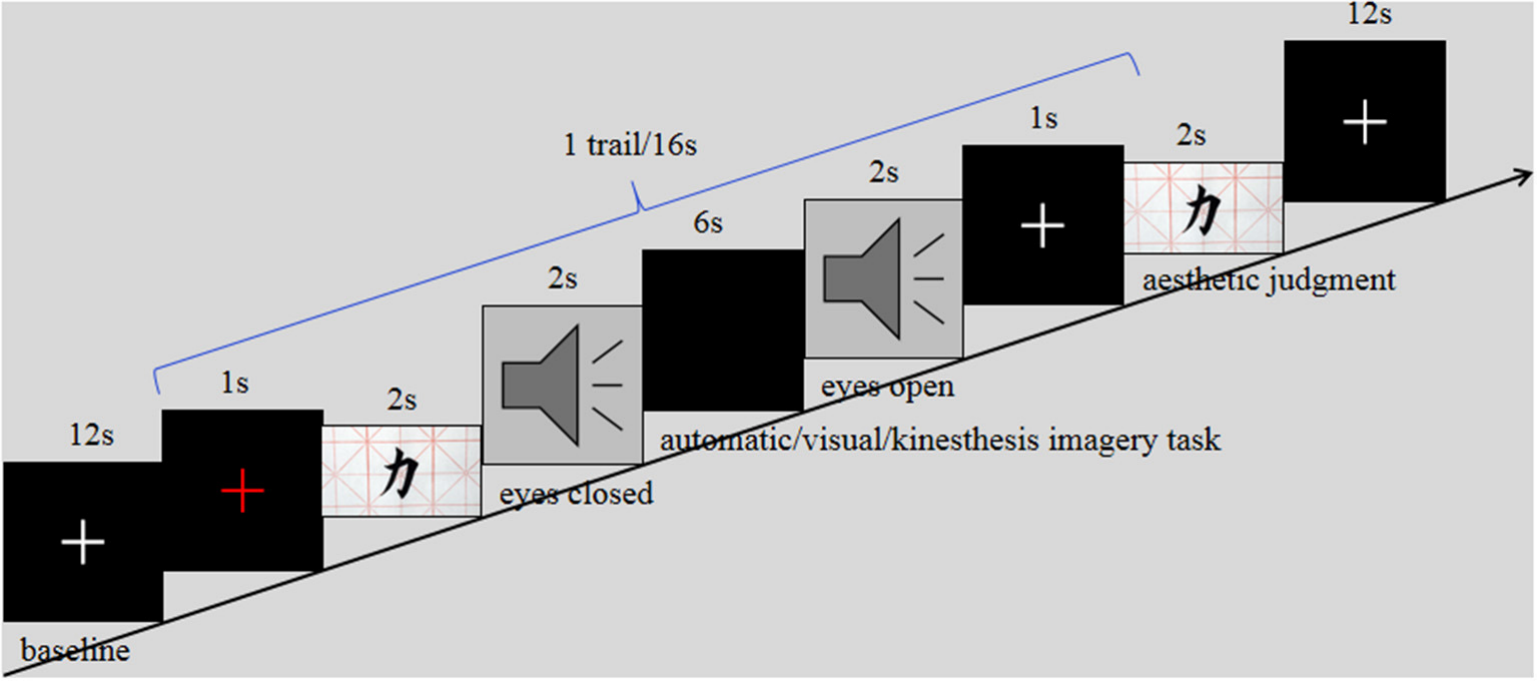
Experimental design, procedure, and examples of stimuli. Three types of tasks were performed in separate runs, namely, kinesthetic, visual, and automatic imagery (aesthetic judgments).

The presentation of all images (resolution: 534 × 300 pixels) was controlled by the E-prime 2.0 (Schneider et al., 2002) and displayed on a liquid-crystal display projector (resolution: 1,920 × 1,080 pixels, 60 Hz refresh rate) at a distance of about 100 cm to the eyes of a participant, covering a visual angle of approximately 4° vertical × 8° horizontal. The scanning session of each participant consisted of three separate run procedures, each corresponding to a motor imagery task, e.g., run1^#^ for the kinesthetic imagery task, run2^#^ for the visual imagery task, and run3^#^ for the BC task. We used a block design with 24 trials which were presented in a random order for each run procedure. Each trial lasted for 16 s. First, the participants were required to keep their eyes fixed onto a cross in the middle of the screen for 1 s, and then one calligraphy image was randomly presented on the screen for 2 s. Later, a tone was presented which lasted for 2 s (refer to Kilintari et al., 2016). According to the instruction, the participants had to close their eyes and perform motor imagery tasks. In the kinesthetic imagery task (run1^#^), they imagined themselves performing the actions to generate the presented calligraphy images (from the perspective of the first person). In the visual imagery task (run2^#^), participants imagined the calligraphy creator of the presented calligraphy images acting as upon creation (from the perspective of the third person). In the BC task (run3^#^), participants closed their eyes and automatically imagined these actions only through the brushstrokes of the calligraphy images (Freedberg and Gallese, 2007; Taylor et al., 2012). Furthermore, 6 s later (the pilot study showed that it lasted for about 6 s for participants to imagine the complete action of writing a character), the participants heard another tone that indicated they should open their eyes quickly and report the preferences of each of the presented images (1 = *I do not like it at all* and 3 = *I like it very much*) in 2 s. Finally, there was a 12-s fixation interval [as a rest condition (RC)]. The order of imagery conditions was counterbalanced across participants. To reduce the fatigue effect, the participants rested for 5 min between two blocks, as shown in Figure 1.

### Instructions for Motor Imagery Task

In the kinesthetic/visual imagery task, the participants were instructed to imagine to create each calligraphy image as follows: “We want you to focus on the calligraphy image as much as possible, and imagine yourself creating this calligraphy image/imagine the calligraphy creator creating this image, for 6 s, from the first stroke to the end of the last stroke.” In the BC task, participants were instructed to do as follows: “We want you to focus on the calligraphy images as much as possible and close your eyes for 6 s.”

### Data Acquisition

The MRI data were collected using a 3T Siemens Prisma_Fit scanner with a 20-channel phased-array head coil at the Magnetic Resonance Imaging Lab, South China Normal University, Guangdong, China. A gradient echo-planar imaging sequence was used with the following parameters: slice thickness = 3 mm, interslice gap = 1 mm, echo time (TE) = 30 ms, repetition time (TR) = 2,000 ms, flip angle = 90°, matrix size = 64 × 64, field of view = 192 mm, and 32 axial slices that covered the whole brain. The T1-weighted three-dimensional structural images were acquired by using a magnetization-prepared rapid gradient-echo sequence, TE = 2.52 ms, TR = 2,530 ms, flip angle = 7°, and voxel size = 1 × 1 × 1 mm^3^.

### Data Analysis

The data preprocessing and analysis were performed using SPM8 (https://www.fil.ion.ucl.ac.uk/spm/). For stabilization of magnetization, the first five volumes of each session were discarded. The remaining images were corrected for slice timing, spatially realigned to the first volume for correcting head movements. The other preprocessing steps were as follows: registered to the T1-weighted structural image, a mean image that was created from realigned volumes and spatially normalized to the montreal neurological institute (MNI) echo-planar imaging brain template using nonlinear basis functions, and resampled with a voxel size of 3 × 3 × 3 mm^3^. The normalized functional images were smooth with an isotropic 6-mm full-width half-maximum Gaussian kernel.

At the first-level analysis, a general linear model was applied to the fMRI time series in which stimulus onset was modeled as single impulse response functions, and then convolved with the canonical hemodynamic response function. We modeled four regressors of interest, namely, an aesthetic judgment of kinesthetic imagery (MI_AJ), an aesthetic judgment of visual imagery (VI_AJ), an aesthetic judgment of BC, and RC. The parameters of head movement calculated from the realignment procedure were included in the model as covariates of no interest. A high-pass filter with a cutoff period of 128 s was applied to remove the low-frequency signal drifts.

The contrast images for aesthetic judgments of calligraphy images and baseline were taken to second-level *t*-test and modeled into the flexible factorial analyses. The main interest of this present study was to identify the cortical networks involved in the aesthetic judgments of calligraphy images after kinesthetic/visual/imagery. We first performed the contrasts of “MI_AJ > RC,” “VI_AJ > RC,” and “BC > RC.” Moreover, direct comparisons of “MI_AJ > BC,” “MI_AJ > VI_AJ,” and “VI_AJ > BC” were conducted to investigate differences in neural mechanisms between aesthetic judgments of calligraphy image after kinesthetic/visual/imagery. All results were significant, and a voxel-wise threshold at *p* < 0.05 family-wise error was corrected at the cluster level (cluster estimated with a voxel-level threshold of the uncorrected *p* < 0.001). The threshold for cluster extension was always 20 voxels. The location of foci of activation was presented in the stereotaxic space of the MNI coordinate system.

## Results

### Behavioral Results

#### The Reaction Rate of Aesthetic Judgments

A generalized linear mixed model (binomial) was performed using the R Software (R Core Team, 2019) with the lme4 package (Bates et al., 2015) to examine how motor imagery affects the aesthetic judgment of a participant, with aesthetic judgment as the dependent variable and imagery type (kinesthetic/visual/baseline) as the fixed factors; random intercepts for participants and slopes were allowed to vary in accordance with the imagery type. This model revealed significant effects of motor imagery on aesthetic judgment. The participants preferred calligraphy images after they performed kinesthetic imagery rather than visual imagery (β = 0.583, *SE* = 0.286, *z* = 2.037, *p* < 0.05) (see Table 1).

**Table 1:** Fixed effects from the generalized linear mixed model constructed to examine how the motor imagery affects aesthetic judgment.

**Fixed effects**	**β**	***SE***	***Z***	***p***
Intercept	1.162	0.320	3.635	<0.001
Visual imagery	−0.583	0.286	−2.037	0.042
Baseline condition	−0.215	0.282	−0.763	0.445

*SE, standard error*.

### Functional Magnetic Resonance Imaging Results

#### Aesthetic Judgments of Kinesthetic/Visual Imagery vs. Rest Condition

**Figure 2:**
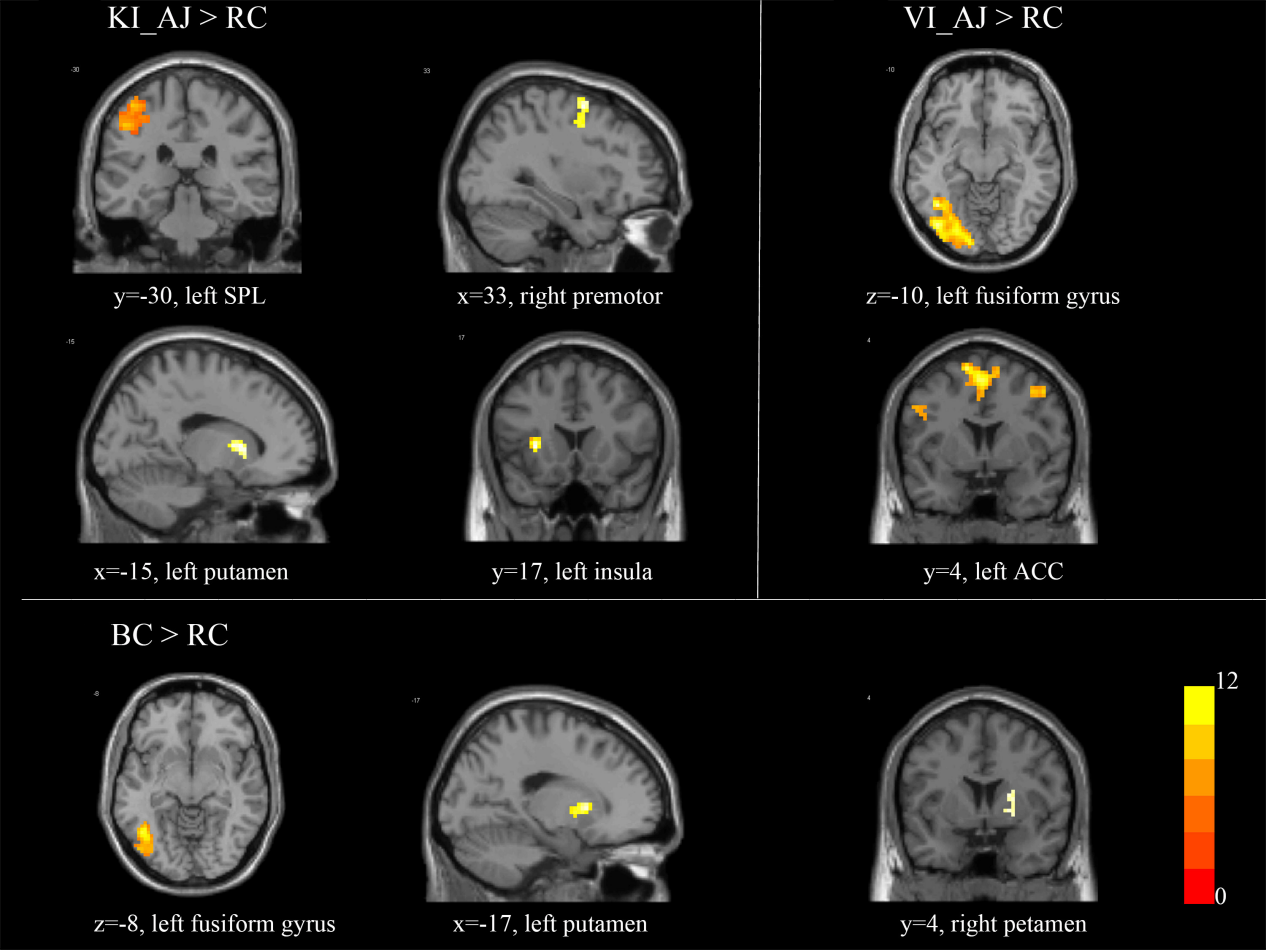
Brain activated during aesthetic judgment for the contrast kinesthetic/visual/automatic imagery vs. baseline. *T-scores* are rendered in colors ranging from 0 (red) to positive (yellow) as indicated by the accompanying colored bars.

In contrast to “MI_AJ > RC,” we revealed a stronger activation in the superior frontal gyrus, the superior parietal lobule, the premotor cortex, the postcentral gyrus, the insula, and the putamen. In contrast to “VI_AJ > RC,” we observed a stronger activation in the bilateral fusiform gyrus, the inferior frontal gyrus, the bilateral middle frontal gyrus, and the ACC. In contrast to “BC_AJ > RC,” we observed the activation of brain regions including the fusiform gyrus, the superior frontal gyrus, the premotor cortex, and the bilateral putamen (see Table 2, Figure 2).

**Table 2:** Brain activated during aesthetic judgment for the contrast kinesthetic/visual/automatic imagery vs. baseline.

**Brain regions**	**Hemisphere**	**Peak MNI coordinates**			***t-score***	**Cluster size**
		**X**	**y**	**z**		
**MI_AJ** **>** **RC**						
Superior frontal gyrus	L	−3	6	60	13.79	183
Superior parietal lobule	L	−24	−57	45	18.17	99
Premotor	R	45	−3	48	10.12	43
Postcentral gyrus	R	48	−30	57	7.98	23
Insula	L	−30	18	3	13.06	24
Putamen	L	−15	6	6	9.18	20
**VI_AJ** **>** **RC**						
Fusiform gyrus	L	−42	−57	−18	14.41	115
	R	27	−63	48	12.68	43
Inferior frontal gyrus	L	−51	6	33	8.29	29
Middle frontal gyrus	R	48	6	48	9.52	32
	L	−27	−3	57	8.17	24
Anterior cingulate cortex	L	−6	6	51	7.51	35
**AI_AJ** **>** **RC**						
Fusiform gyrus	L	−41	−60	−15	15.26	50
Superior frontal gyrus	L	−6	6	54	12.4	67
Premotor	L	−33	−6	54	8.10	34
Putamen	L	−21	9	3	10.72	69
	R	24	0	15	8.11	32

*Coordinates refer to the stereotactic space of the Montreal Neurological Institute (p <0.05, family-wise error [FWE] corrected)*.

*MI_AJ, aesthetic judgment of kinesthetic imagery; RC, rest condition; VI_AJ, aesthetic judgment of visual imagery*.

#### Aesthetic Judgments of Kinesthetic/Visual vs. Baseline Condition

**Figure 3:**
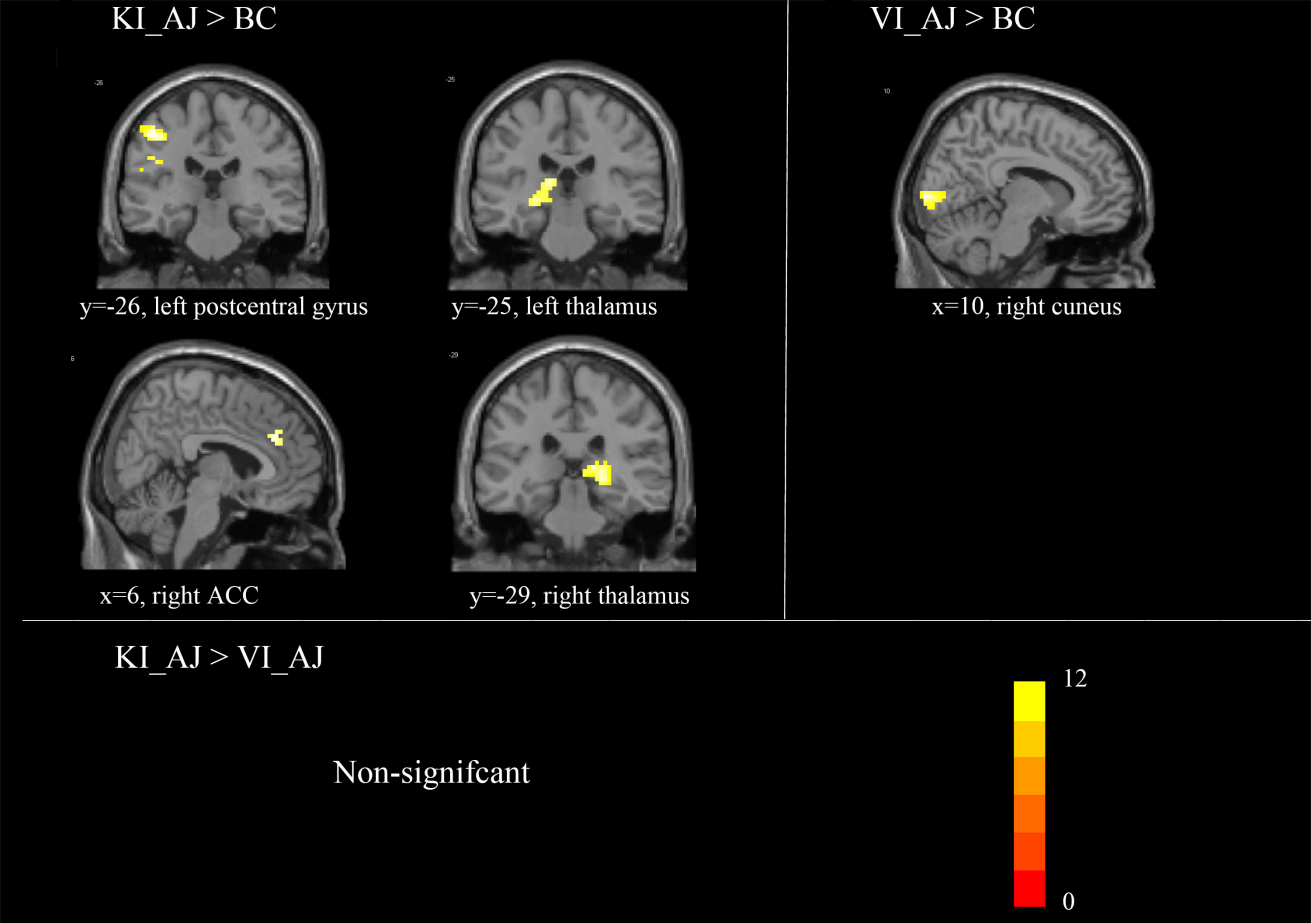
Brain activated during aesthetic judgment for the contrast kinesthetic/visual automatic imagery. *T-scores* are rendered in colors ranging from 0 (red) to positive (yellow) as indicated by the accompanying colored bars.

To investigate the neural mechanism of the aesthetic judgments of motor imageries, a direct comparison was conducted between the aesthetic judgments of kinesthetic/visual/BC. In contrast to “MI_AJ > BC_AJ,” we observed a stronger activation in the middle frontal gyrus, the postcentral gyrus, the ACC, and the thalamus. In contrast to “MI_AJ > VI_AJ,” we revealed no significant activation. In contrast to “VI_AJ > BC_AJ,” we observed stronger activation in the cuneus (see Table 3, Figure 3).

**Table 3:** Brain activated during aesthetic judgment for the contrast kinesthetic/visual/automatic imagery.

**Brain regions**	**Hemisphere**	**Peak MNI coordinates**			***t-score***	**Cluster size**
		**X**	**y**	**z**		
**MI_AJ** **>** **BC**						
Middle frontal gyrus	R	51	15	42	8.96	25
Postcentral gyrus	L	−48	−18	27	10.54	93
Anterior cingulate cortex	R	6	33	27	7.67	12/35
Thalamus	L	−24	−27	0	8.30	65
	R	21	−30	0	8.83	91
**VI_AJ** **>** **BC**						
Cuneus	R	9	−93	3	9.8	48
**MI_AJ** **>** **VI_AJ**						
Non-signifcant						

*Coordinates refer to the stereotactic space of the Montreal Neurological Institute (p <0.05, family-wise error [FWE] corrected)*.

*BC, baseline condition; MI_AJ, aesthetic judgment of kinesthetic imagery; VI_AJ, aesthetic judgment of visual imagery*.

## Discussion

The present study used Chinese calligraphy as materials to explore the impact of imagery on the action of a creator on the aesthetic judgment of participants. The behavioral results indicated that, compared with automatic imagery and visual imagery, there was a tendency for kinesthetic imagery to promote the aesthetic judgment of participants. In our results of neuroimaging, we found that the fusiform gyrus was activated for aesthetic judgments of Chinese images in contrast to both “VI_AJ > RC” and “BC_AJ > RC.”

In general, the previous neural studies have demonstrated that the perceptual processing of beauty is associated with the activation of the fusiform gyrus, a region relevant to visual preference and attention modulation (Zhang et al., 2017). Furthermore, prior studies have revealed that the subdivisions of the fusiform gyrus encoded images of human bodies (Peelen and Downing, 2005; Schwarzlose et al., 2005; Di Dio et al., 2016). In the visual imagery task, participants imagined the creative actions of others as spectators from the perspective of the third-person perspective. Meanwhile, Freedberg and Gallese (2007) proposed that observers could unconsciously imagine the gestures of the creators, which were provoked by brushstrokes. Thus, the activation of the fusiform gyrus in this study probably reproduced a complete visual analysis of the physical features of the body (Di Dio et al., 2016). However, it should be noted that the fusiform gyrus, which is frequently defined as the region of visual word forms, might also be involved in the processing of reading single words (Jobard et al., 2003).

Furthermore, we also observed that the superior frontal gyrus and inferior frontal gyrus were activated in three types of imagery tasks. Previous neuroimaging results revealed that the frontal cortex was mainly involved in cognitive judgment and emotional handling (Boccia et al., 2016; Zhang et al., 2016). For example, Zhang et al. (2016) found that the bilateral inferior frontal gyrus was activated for aesthetic judgments of beauty for both the pictographs and the object images. Yeh et al. (2015) also found that both the subjective ugliness and negative emotion triggered the inferior frontal gyrus. Also, the middle frontal gyrus and the inferior gyrus that were triggered may be engaged in language processing. For example, the large parts of the frontal lobe were triggered when the participants were demanded to read numerous proverbs (Bohrn et al., 2013), poetry (Gao and Guo, 2018), and prose (Zeman et al., 2013). These findings indicated that the process of appreciating Chinese calligraphy involved the perception of the visual words to a minimum extent.

Moreover, we found that the superior parietal lobule, the premotor cortex, and the postcentral gyrus were activated in contrast to “MI_AJ > RC”; these regions were also recruited as a core network in motor imagery (see a meta-analysis by Hardwick et al., 2018). Therefore, we argued that the activation of these regions indicated that the participants successfully felt the movement and perceived muscle contractions and stretched mentally from the perspective of the first person. In other words, the participants effectively carried out the task of imagining themselves performing a calligraphic creation.

Furthermore, an earlier neuroimaging research proposed that the putamen (Huang et al., 2016; Eugen et al., 2017; Zhang et al., 2017), the insula cortex (Vartanian and Skov, 2014; Gao and Guo, 2018), the ACC (Sarita et al., 2015; Yeh et al., 2015; Ishizu and Zeki, 2017), and the thalamus (Kühn and Gallinat, 2012; Elvira et al., 2016) were engaged in the processing of aesthetic emotions. In line with the previous studies, we reported the activation of these brain regions in our imaging results, which suggested that the participants gained positive aesthetic experience after performing the motor imagery task. For example, we observed the activation of the insula and the putamen in contrast to “MI_AJ > RC.” The two meta-analyses have revealed that the insula was recognized to perform a critical role in emotional processing (e.g., hedonic) and was composed of the part of the significant effective structure of the brain (Vartanian and Skov, 2014; Zou et al., 2016). Moreover, we also observed the activation of the putamen in contrast to “AI_AJ > RC”; this structure was regarded as the subcortical reward region located in the basal ganglia (Liu et al., 2011; Wang et al., 2015; Eugen et al., 2017; Zhang et al., 2017). Vartanian and Skov (2014) proposed that the activation of the insula cortex and the putamen possibly involved a conscious evaluation during the later stages of the processing of aesthetic information.

More importantly, we observed the activation of the ACC in contrast to both “VI_AJ > RC” and “MI_AJ > BC.” Numerous previous studies have shown that the ACC could be activated through the appreciation of natural landscapes (Zhang et al., 2019), joyful beauty (Ishizu and Zeki, 2017), painting (Sarita et al., 2015), subjective beauty (Yeh et al., 2015), and eye-catching faces (see a meta-analysis by Hu et al., 2020). A general activation likelihood estimation meta-analysis by Boccia et al. (2016) demonstrated that the ACC played a critical role in features of the so-called “aesthetic state of mind,” which inspired the handling of emotional features of aesthetic-related reactions and their incorporation with the higher-order cognitive assessment. Interestingly, we also observed the thalamus in contrast to “MI_AJ > BC,” which was considered as an important value coding center for reward experience. For example, a meta-analysis of neuroimaging studies by Sescousse et al. (2013) found that the thalamus was one of the structures consistently related to diverse monetary, food, and erotic rewards. Therefore, the activation of the ACC and the thalamus indicated that compared with automatic imagery, participants gained more positive aesthetic experience after performing the kinesthetic imagery task. Notably, the perception and appreciation of calligraphy images involved the cuneus activation in contrast to “VI_AJ > BC,” which is consistent with the verdicts of Zhang et al. (2017) that higher activation in the cuneus was perceived during the judgment of beauty in pictographs. In fact, as part of the visual structure, the activation of the cuneus also reflects the aesthetic preference of the participants. For example, prior neuroimaging studies have shown that the cuneus was associated with the aesthetic judgment of representative paintings (Mizokami et al., 2014; Yoshinori et al., 2014). Therefore, the activation of the cuneus indicated that the participants had a more positive aesthetic experience after performing the visual imagery task. As mentioned earlier, active imagery, compared with automatically imagining the creative action of the artist through clues, is more likely to involve deeper aesthetic processing when appreciating Chinese calligraphy. Meanwhile, based on the above discussion, the neuroimaging results confirmed our previous hypotheses.

## Limitations

The study had certain limitations. First, we failed to find the behavioral difference. Here are some possible reasons. On the one hand, we used forced selection (liking versu disliking) rather than a 7-point or 9-point Likert scale to ask the participants to make aesthetic judgments. As a result, this may lead to difficulties in detecting small behavioral differences out of the three imagery types. In contrast, since we collected only the fMRI data from 20 participants, such a small sample size may have reduced the statistical power of this study in the behavioral data. It may also cause the failure to observe the brain region in contrast to “MI_AJ > VI_AJ” activation. In addition, although behavioral results have suggested a statistical difference between motor imagery and visual imagery, we failed to find any meaningful brain differences between these two conditions. One possible way was to combine these two conditions to explore the effects of motor imagery on brain activation, while we found technical difficulties in processing the data due to the experimental design if we combined these two conditions. Second, there was no objective indicator to measure whether the participants effectively performed the imagination tasks, which possibly affected the results. Third, using cross-fixation as a BC might not effectively control the activity in motor brain regions associated with the key responses. Finally, it should be noted that although we have matched on many properties among the three groups of materials, we did not counterbalance these stimuli across participants, which might cause the condition differences and further impact the results.

## Conclusion

In line with prior studies, an aesthetic judgment of Chinese calligraphy is involved in perceptual processing, cognitive judgment, emotional, and reward processing. This study provided empirical evidence that, compared with automatic imagery elicited by clues, such as brushstrokes, actively performing motor imagery tasks, especially kinesthetic imagery, influenced affective responses to art. This study contributed to the behavioral and neural mechanisms of aesthetic appreciation involved in the visual art.

## Data Availability Statement

The original contributions presented in the study are included in the article/supplementary material, further inquiries can be directed to the corresponding author.

## Ethics Statement

The studies involving human participants were reviewed and approved by the Institute Ethics Committee, South China Normal University. The patients/participants provided their written informed consent to participate in this study.

## Author Contributions

MH and XH designed the experiments and drafted the article. WZ, HS, and YL revised the manuscript critically. XL, YD, and HW contributed to data preprocessing, data collection, and making experimental materials. All authors contributed to the study and approved the submitted version.

## Conflict of Interest

The authors declare that the research was conducted in the absence of any commercial or financial relationships that could be construed as a potential conflict of interest.

## Publisher's Note

All claims expressed in this article are solely those of the authors and do not necessarily represent those of their affiliated organizations, or those of the publisher, the editors and the reviewers. Any product that may be evaluated in this article, or claim that may be made by its manufacturer, is not guaranteed or endorsed by the publisher.
